# A Method for Identifying Hydration Stages of Concrete Based on Embedded Piezo-Ultrasonic Active Sensing Technology

**DOI:** 10.3390/ma18204722

**Published:** 2025-10-15

**Authors:** Min Xiao, Yaoting Zhu, Wei Min, Feilong Ye, Yongwei Li, Xunhao Ding, Tao Ma

**Affiliations:** 1 Jiangxi Communications Investment Group Co., Ltd., No. 367, Chaoyangzhou Middle Road, Nanchang 330025, China; xiaom123555@163.com (M.X.); weim880905@163.com (W.M.); liyw123876@163.com (Y.L.); 2 Jiangxi Communications Investment Maintenance Technology Group, No. 809, Jinsan Avenue, Xiaolan Economic Development Zone, Nanchang 330052, China; 3 School of Transportation, Southeast University, Nanjing 211186, China; yfl9620@seu.edu.cn (F.Y.); matao@seu.edu.cn (T.M.)

**Keywords:** piezoelectric sensing, piezoelectric wave propagation method (PWPM), concrete hydration, penetration resistance, compressive strength, setting time

## Abstract

The structural evolution of concrete during different hydration stages critically influences subsequent strength, and continuous monitoring throughout this process has become a research focus in materials science. This study proposes an embedded ultrasonic active sensing technique based on piezoelectric ceramics (PZT) to identify key structural transition stages during concrete curing. To this end, a piezoelectric ultrasonic sensor was fabricated and its comprehensive performance was systematically evaluated. Subsequently, compressive strength and penetration resistance tests were conducted, and the evolution of piezoelectric signal amplitude and wavelet packet energy (WPE) during hydration was analyzed. Furthermore, a root mean square deviation index based on WPE (WPE-RMSD) was introduced to identify structural transitions throughout the hydration process. The results demonstrate that the developed sensor exhibits stable electrical, mechanical, and waterproof performance. Both signal amplitude and WPE effectively captured the hydration process of concrete, with WPE showing higher sensitivity. The WPE-RMSD index exhibited good temporal continuity, covering the entire process from early hydration disturbance to late-stage structural densification (28 d), and proved particularly effective in identifying critical stages such as final setting and the medium-age period (7 d). This study provides a novel in situ monitoring approach for the classification and identification of hydration stages in concrete.

## 1. Introduction

The hydration process of cement-based materials is a fundamental physicochemical evolution that governs the early development of strength, stiffness, and long-term durability of concrete [1,2,3]. During this process, concrete transforms from a fluid suspension into a solid load-bearing material, accompanied by microstructural changes such as nucleation, growth, particle packing, and pore structure refinement. In particular, the setting and hardening stages are highly sensitive to environmental conditions and mix proportions, leading to significant variations in setting time and curing progression under different water-to-cement ratios and curing regimes, which directly influence the subsequent strength development and structural performance [4,5,6].

At the laboratory scale, standard testing methods can be employed to evaluate the early hydration process of concrete [7,8]. However, these methods typically lack the capability for continuous monitoring of the evolving material properties. As a result, real-time and non-destructive monitoring of the cement hardening process has become a key research focus in materials science. Over the past decades, various non-destructive techniques have been developed to characterize cement hydration, including scanning electron microscopy [9], isothermal calorimetry [10], chemical shrinkage measurements [11], electrical conductivity [12,13] and pH monitoring [14]. Vipulanandan et al. [15] used electrical resistivity measurements to investigate the evolution of cement-based materials under different curing conditions. Hong et al. [16] applied the electrodeless resistivity method to monitor the early hydration of Magnesium Potassium Phosphate Cement (MKPC), establishing a relationship between setting time and resistivity inflection points, and proposed a linear model to predict 28-day compressive strength based on 24 h resistivity. Silva et al. [17] employed alternating current impedance spectroscopy (AC-IS) to assess the effects of nanomaterials on cement mortar, demonstrating its effectiveness in monitoring microstructural evolution. Lin et al. [18] used AC impedance spectroscopy (ACIS) for long-term, non-destructive evaluation of concrete, revealing correlations between resistivity, degree of reaction, chemical shrinkage, and compressive strength. Guo et al. [19] proposed a novel non-destructive method combining low-field nuclear magnetic resonance and cyclic voltammetry and found that strength-weighted relaxation time and specific capacitance could indicate the degree of hydration. Additionally, recent studies have explored the feasibility of using Raman spectroscopy-based sensor systems for monitoring the early hydration of fresh cement paste, confirming its capability for real-time and continuous tracking of long-term clinker hydration processes [20,21].

Although various testing techniques have been employed to characterize the hydration process of cement-based materials, most still face inherent limitations. Chemical and physical measurements provide macroscopic insights into reaction kinetics and volumetric changes but typically rely on destructive sampling or intermittent testing, making it difficult to achieve continuous monitoring throughout the hydration process. Electrical methods, such as resistivity and AC impedance spectroscopy, can reflect ion transport and reaction kinetics; however, their responses are highly sensitive to environmental factors (e.g., humidity, temperature, and electrode contact), which compromises their long-term stability and accuracy. Spectroscopic techniques enable microstructural characterization at the molecular or pore scale but generally require complex experimental setups and expensive precision instruments, limiting their applicability in field monitoring. Therefore, despite the diversity of existing nondestructive testing techniques, there remains a lack of approaches capable of achieving real-time, in situ, and quantitative monitoring of the early hydration process.

With the advancement of intelligent monitoring technologies for concrete, PZT sensors—serving as active sensing devices based on electromechanical impedance (EMI) and piezoelectric wave propagation methods (PWPM) —have attracted increasing attention for monitoring the early hydration of cement-based materials [22]. Narayanan et al. [23] developed an embedded PZT sensing system, demonstrating that the impedance response can sensitively capture material state transitions and mechanical impedance evolution during setting and early strength development stages, while maintaining long-term durability. Li et al. [24,25] applied the EMI technique to monitor the setting behavior of fresh cement paste and found that reliable signals could be obtained within the first 12 h, facilitating more accurate determination of setting time. Kaur et al. [26] proposed a novel method for in situ monitoring of early-age strength in rapid-setting concrete, utilizing the EMI resonance peak parameters of embedded resin-coated piezoelectric sensors to track the curing process. Yang et al. [27] designed a new smart piezoelectric module, establishing a mortar strength prediction model by extracting features from pulse wave signals, which demonstrated high fitting accuracy across concrete at different ages. Jiang et al. [28] further integrated embedded SPM with elastic wave theory and proposed a wave modulus index derived from wave velocity, enabling real-time strength prediction during the curing process of mortar and concrete. In addition, Haq et al. [29] systematically compared the monitoring performance of EMI and PWPM over the 1–28 day curing period, showing that the electrical RMSD extracted from EMI exhibited higher sensitivity to hydration reactions than changes in P-wave velocity. In summary, piezo-ultrasonic based sensing methods can effectively capture structural evolution features during early cement hydration, providing significant technical support for structural health monitoring of concrete structures [30,31].

In summary, early hydration monitoring of cement-based materials has largely relied on EMI-based sensing techniques, which can reflect the densification of the microstructure and the development of mechanical strength through conductivity or impedance spectra. Compared with EMI, PWPM not only captures the propagation velocity of ultrasonic waves within the material but also provides information on energy attenuation and dispersion characteristics, offering richer insights into structural evolution and demonstrating significant potential for detecting microstructural transitions during hydration. It is noteworthy that most existing studies often overlook the durability evaluation of piezoelectric sensors and do not systematically denoise the raw signals during acquisition and analysis. This may result in subtle structural changes being obscured by noise, thereby limiting the accurate identification of the setting and structural evolution stages of concrete.

In view of the aforementioned limitations, this study proposes an innovative monitoring method based on energy evolution characteristics. By integrating WPE and RMSD features, an Energy Deviation Response Index (EDRI) was established to quantify the deviation of cumulative signal energy, thereby identifying critical setting and structural transition points during the hydration process. To validate the effectiveness of this method, embedded PZT sensors were encapsulated and protected using cement mortar, and their overall performance was systematically evaluated. A piezoelectric ultrasonic sensing system was constructed to ensure stable excitation and high-fidelity reception of ultrasonic signals within the concrete. Concrete specimens with three different water-to-cement ratios (*w*/*c* = 0.40, 0.45, and 0.50) were prepared to simulate strength evolution under varying hydration rates. Reference strength indices were obtained via penetration resistance and standard compressive strength tests. Signal denoising techniques were applied to the acquired data for pre- and post-processing, WPE features were extracted, and the RMSD metric was introduced to quantify energy deviation patterns. This study provides a novel pathway for early-age concrete performance monitoring based on PWPM.

## 2. Experimental Program

### 2.1. Materials and Preparation

#### 2.1.1. Concrete Materials and Specimen Preparation

**Figure 1:**
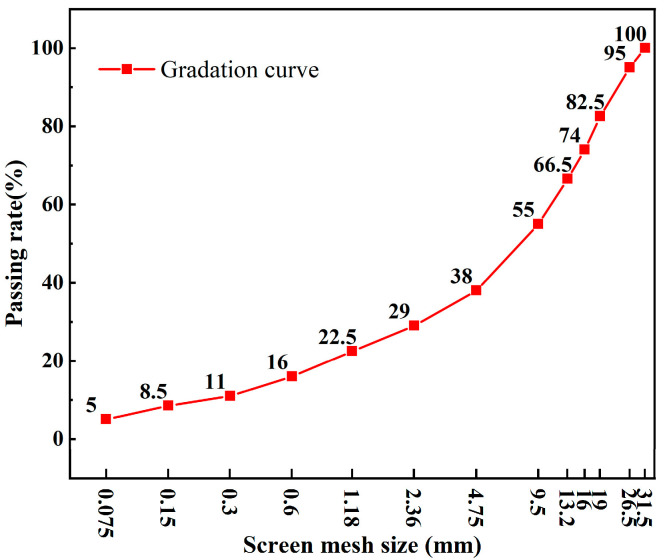
Aggregate gradation curve.

To evaluate the accuracy of piezoelectric ultrasonic sensors in monitoring early strength development of concrete with varying *w*/*c*, three concrete mixtures with *w*/*c* ratios of 0.40, 0.45, and 0.50 were prepared. The mix design was based on a target density of 2350 kg/m^3^, as detailed in Table 1. The cement used was P.O 42.5 ordinary Portland cement, and the coarse aggregate was natural high-calcium limestone, with its particle size distribution shown in Figure 1. The fine aggregate was natural river sand with a particle size of 0–1.5 mm, and the admixture was ZY-HPWR-S type polycarboxylate high-performance water reducer. For each mixture, eighteen 100 mm × 100 mm × 100 mm cubic specimens were cast for compressive strength tests. In addition, three specimens were prepared for each group to evaluate the penetration resistance during the early hydration stage. All specimens were cured under standard conditions, specifically at a temperature of 20 ± 2 °C and a relative humidity of ≥95%.

#### 2.1.2. Piezoelectric Sensor and Materials

Considering the excellent electromechanical coupling coefficient and piezoelectric strain constant of PZT-5A, a pair of commercial flat piezoelectric ceramic disks (15 mm in diameter and 1 mm in thickness) were employed as ultrasonic transducers. The material properties (Table 2) are derived from the product specifications provided by the manufacturer. To ensure stable signal transmission and reception of the sensor, a shielded wire (with a diameter of 3.9 mm) with high-temperature resistance properties was used for electrical connection. For the external encapsulation and protection of the sensor, polymer waterproof mortar was selected to enhance its impermeability and structural strength.

#### 2.1.3. Fabrication of the Sensor Specimens

**Figure 2:**
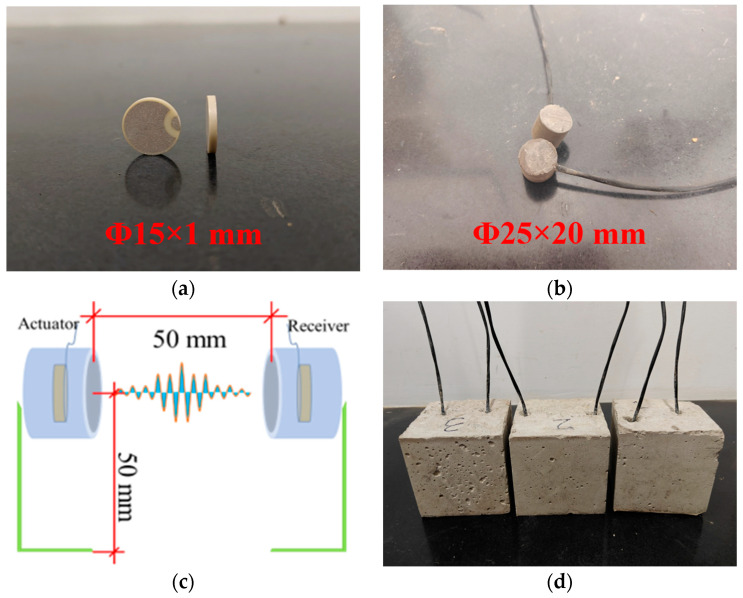
Encapsulation process of the piezoelectric ultrasonic sensor: (**a**) PZT element; (**b**) Piezoelectric ultrasonic sensor; (**c**) Sensor fixation diagram; (**d**) Specimens.

First, two clean and intact piezoelectric ceramic discs were selected as the sensing elements, as shown in Figure 2a. Subsequently, a shielded wire was used to electrically connect the two piezoelectric ceramics. To prevent short-circuiting, the surface of the PZT was coated with epoxy resin and cured at room temperature for 12 h to ensure solidification. During the encapsulation process, a PE tube with an inner diameter of 25 mm and a height of 20 mm was used as a mold, and polymer waterproof mortar was chosen as the encapsulation material. After the encapsulation material was poured, it was compacted by vibration and left to stand for 24 h before demolding. Finally, the sensors were cured at room temperature for 28 d to ensure its structural stability and encapsulation integrity. The finished appearance of the sensor is shown in Figure 2b.

Each sensor was mounted on an iron plate with a 90° bend to ensure a fixed height of 50 mm and secured at the bottom of the mold. The center-to-center spacing between the transmitter and receiver was 50 mm, as shown in Figure 2c. Based on the designed concrete mixtures, three types of specimens with embedded piezoelectric ultrasonic sensors were fabricated for wave propagation analysis, as shown in Figure 2d.

### 2.2. Sensor Performance Evaluation

Capacitance is a key parameter of the electrical performance of piezoelectric ultrasonic sensor, reflecting the material’s ability to store charge under an applied electric field. Therefore, the magnitude of capacitance directly affects the output signal and response characteristics of the piezoelectric sensor. During the manufacturing and encapsulation process of piezoelectric ceramics, the capacitance value may undergo significant changes, as noted in the study by Elvin N. et al. [32]. To this end, a multimeter (ZTW-890C, Chint Electrics, Dongguan, China) was used to measure the capacitance of six PZT-5A piezoelectric discs under different operating conditions. The specific conditions are: Condition 1—Piezoelectric ceramics connected to the shielded wire; Condition 2—Waterproof treatment of the piezoelectric ceramics using epoxy resin; Condition 3—Immersion of the sensor in water for 15 min; Condition 4—Encapsulation using polymer waterproof mortar; Condition 5—Curing of the encapsulated piezoelectric sensor for 28 d.

The mechanical and waterproof properties of the piezoelectric ultrasonic sensor have a significant impact on its sensitivity. To evaluate the mechanical performance of the sensors, three different piezoelectric ultrasonic sensors were tested using a universal testing machine after 28 days of standard concrete curing, following the Chinese National Standard GB/T 1041-2008 [33]. In addition, to evaluate the impermeability of the encapsulation layer, three groups of sensors were fully immersed in deionized water, and monthly removed for cleaning, weighing, and capacitance measurement to monitor their long-term waterproof performance.

### 2.3. Mechanical Properties Testing

#### 2.3.1. Penetration Resistance Test

**Figure 3:**
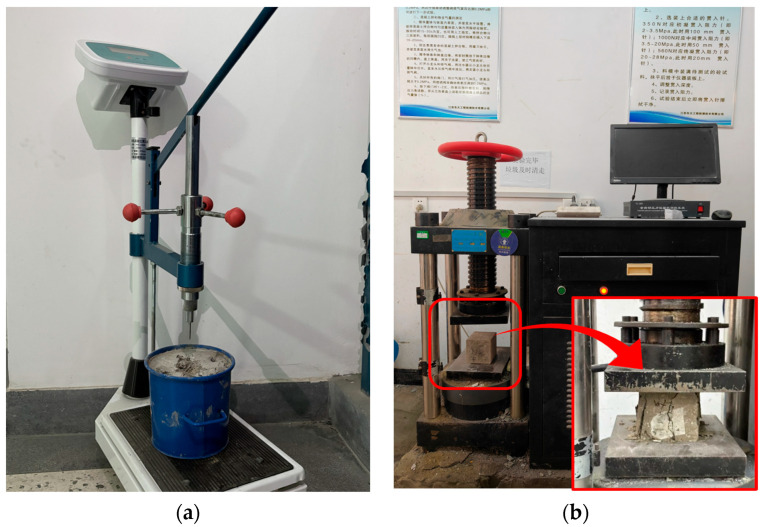
Strength testing devices: (**a**) Penetration resistance testing apparatus; (**b**) Compression testing machine.

The penetration resistance test is a commonly used method to evaluate early strength development and setting time of concrete [34]. In this study, the test was conducted in accordance with the guidelines specified in JTG 3420-2020 [35]. During testing, a probe with a fixed cross-sectional area was vertically driven into the concrete specimen at a constant rate. The applied force required to achieve a penetration depth of 25 mm was recorded, and the penetration resistance per unit area was calculated using Equation (1). The probe dimensions are listed in Table 3. For each specimen, one to two measurement points were selected, and the arithmetic mean was taken as the representative resistance value. Measurements began 2 h after mixing, with intervals of 0.5 h during the early stage and 1 h after 8 h. The testing procedure is illustrated in Figure 3a.

(1) $$f_{PR}=\frac{P}{A}$$

where *f_PR_* is the penetration resistance per unit area (MPa); *P* is the applied force (N) required to reach a penetration depth of 25 mm; *A* is the cross-sectional area of the penetration probe (mm^2^).

#### 2.3.2. Uniaxial Compression Test

Uniaxial compression tests were conducted on concrete specimens at various curing ages in accordance with JTG 3420-2020 [35]. The demolded cubic specimens were cured under standard conditions until 1, 3, 5, 7, 14, and 28 days, and compressive strength tests were conducted at each corresponding age. The loading was performed using a DYE-2000 compression testing machine (Beijing Beifang, Beijing, China) at a constant rate of 5 kN/s until failure. The average compressive strength of the three specimens was reported as the representative value for each age.

### 2.4. PWPM Measurement

#### 2.4.1. Piezoelectric Active Sensing Monitoring System

**Figure 4:**
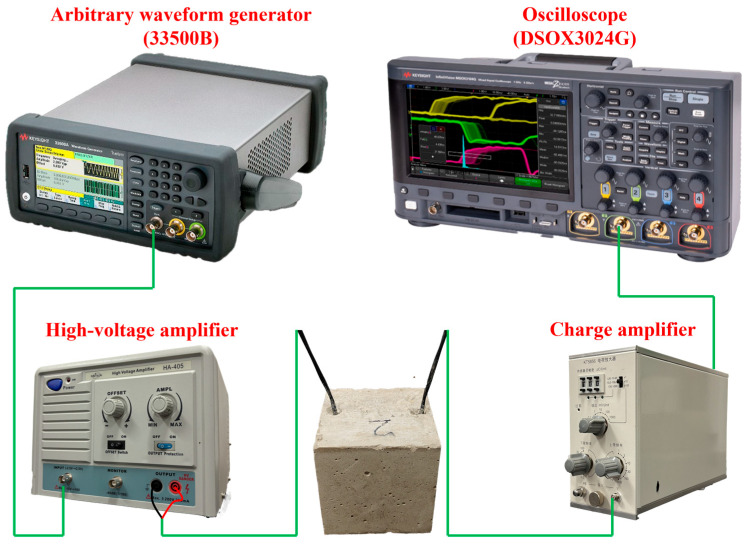
Piezoelectric testing apparatus.

Simultaneously with the penetration resistance and compressive strength tests, PWPM-based piezoelectric ultrasonic monitoring was conducted [36]. Based on the existing research results [27,37,38], this study monitored a single concrete specimen under the same water-cement ratio conditions, with a focus on the evolution pattern of the PZT signal during the hydration process. A pair of embedded PZT-5A sensors (Morgan Electro Ceramics, Bedford, OH, USA) was used as the actuator and receiver. The excitation signal, generated by a 33500B arbitrary waveform generator (Keysight Technologies, Santa Rosa, CA, USA), consisted of a five-peak wave with five cycles and a voltage amplitude of 1 V, repeated at 100 kHz to match the first resonant frequency of the PZT-5A. The signal was amplified 100 times by a high-voltage amplifier and applied to the actuator, while the received waveform was recorded in real time using the DSOX3024G oscilloscope (Keysight Technologies, Santa Rosa, CA, USA) and the charge amplifier. A schematic diagram of the PWPM setup is shown in Figure 4. Measurements began 2 h after casting and were recorded every 30 min. From 8 h to 13 h, the interval was adjusted to 1 h. Additional measurements were performed at 1, 3, 5, 7, 14, and 28 d to capture the structural evolution of concrete with different water-to-cement ratios.

#### 2.4.2. Front-End Signal Processing

During the transmission and reception of signals by the piezoelectric ultrasonic sensor, inevitable environmental noise leads to distortion of the raw electrical signal. To address this issue, the raw time-domain signal *x*(*n*) was first transformed into the frequency domain using the Fast Fourier Transform (FFT), as expressed in Equation (2), to analyze its spectral components.

(2) $$X\left(f\right)=\sum_{n=0}^{N-1}x(n)\cdot e^{-j2\pi fn/N}$$

where *X*(*f*) denotes the frequency-domain representation of the signal, *x*(*n*) is the time-domain signal corresponding to the sampling point *n*, *N* is the total number of sampling points, and *f* represents the frequency.

The frequency spectrum was then examined to identify the range of clutter frequencies. Based on this analysis, a band-pass filter was designed and applied to suppress unwanted frequency components while preserving the target signal, with the center frequency set to 100 kHz and the bandwidth to 100 kHz. The frequency response of the band-pass filter, *H*(*f*), is given in Equation (3).

(3) $$H(f)=\frac{1}{1+{\left(\frac{f}{f_{high}}\right)}^{2n}}\cdot \frac{1}{1+{\left(\frac{f_{low}}{f}\right)}^{2n}}$$

where *f_low_* and *f_high_* represent the lower and upper cutoff frequencies of the filter, respectively, while *n* denotes the order of the filter, which controls the steepness of the filter response.

The filter was applied to the spectral signal *X*(*f*) through frequency-domain convolution to obtain the filtered signal *X_filtered_*(*f*):

(4) $$X_{filtered}\left(f\right)=X\left(f\right)\cdot H\left(f\right)$$

Finally, the Inverse Fourier Transform (IFFT) was employed to convert *X_filtered_*(*f*) back into the time-domain signal *x_filtered_*(*n*):

(5) $$W_{j,k}=\sum_{f=0}^{N-1}x_{filtered}(n)\cdot \psi_{j,k}(n)$$

#### 2.4.3. Back-End Signal Processing

To further enhance signal purity, the Discrete Wavelet Transform (DWT) was introduced to perform multi-scale analysis and denoising of the filtered piezoelectric signal *x_filtered_*(*n*). Figure 4 illustrates the comparison between the original and denoised time-domain signals. First, the time-domain signal was decomposed into a series of low- and high-frequency sub-signals using wavelet basis functions, as expressed in Equation (6):

(6) $$\;W_{j,k}=\sum_{f=0}^{N-1}x_{filtered}(n)\cdot \psi_{j,k}(n)$$

where *W_j_*_,_*_k_* denotes the wavelet coefficient at the *j*-th decomposition level, and *ψ_j_*_,_*_k_*(*n*) represents the wavelet basis function at scale *j* after translation and dilation.

In the denoising procedure, the Daubechies 4 (db4) wavelet was selected as the mother wavelet, and a three-level decomposition was employed to balance time-frequency resolution. The high-frequency detail coefficients were processed using a soft-thresholding method to suppress noise, as given in Equation (7):

(7) $${\hat{W}}_{j,k}=\mathrm{sign}(W_{j,k})\cdot \max(|W_{j,k}|-\lambda ,\;0)$$

The threshold *λ* was determined according to the statistical characteristics of the detail coefficients, expressed as:

(8) $$\lambda =3\cdot \mathrm{std}(D_{j})$$

where std(*D_j_*) denotes the standard deviation of the detail coefficients at level *j*.

Finally, the denoised coefficients were reconstructed into the time-domain signal via the Inverse Discrete Wavelet Transform, as shown in Equation (9):

(9) $${\hat{x}}_{filtered}(n)=\sum_{j,k}{\hat{W}}_{j,k}\cdot \psi_{j,k}(n)$$

where ${\hat{x}}_{filtered}(n)$ represents the denoised time-domain signal.

**Figure 5:**
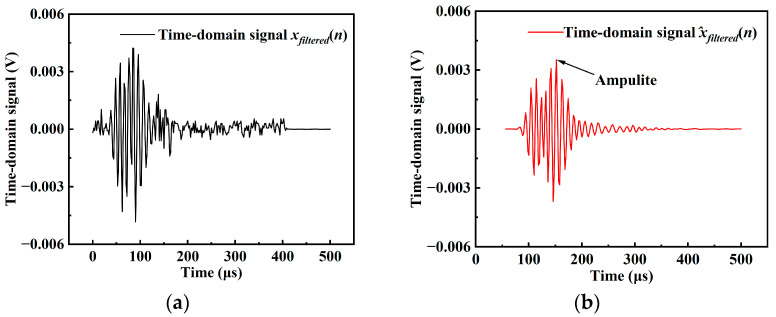
Comparison between the original and denoised time-domain signals: (**a**) original signal; (**b**) denoised signal.

As shown in Figure 5, after the aforementioned front-end and back-end signal processing, the processed data exhibit a significantly improved signal-to-noise ratio compared with the original signal, and the waveform features become more distinct and recognizable.

#### 2.4.4. Signal Feature Extraction and Analysis

Signal feature extraction is a critical step in the quantitative analysis of cement hydration, as it reduces the dimensionality of complex signals while preserving information closely related to structural performance. In particular, the amplitude of the time-domain signal, which directly reflects energy attenuation and nonlinear effects, is highly sensitive to variations in the elastic modulus of concrete and thus serves as an important criterion for distinguishing different stages of cement hydration [37,39,40].

Furthermore, hydration experiments have demonstrated that WPE can serve as an important feature parameter for identifying and monitoring the strength evolution of concrete [41,42]. To validate its effectiveness, ultrasonic propagation tests were conducted on concretes with different water-to-cement ratios at multiple hydration ages. Signal energy features were extracted through wavelet packet analysis and correlated with mechanical properties to assess the feasibility of WPE as a strength-related indicator.

In this study, db4 wavelet was employed as the basis function, and a three-level wavelet packet decomposition was performed. This resulted in eight sub-band signals, from which energy distribution parameters were extracted to characterize the signal variations across different hydration stages. The wavelet packet energy can be represented as an energy vector:

(10) $$E=[e_{1},e_{2},\ldots ,e_{8}]$$

In Equation (10), *e_i_* denotes the energy of the *i*-th sub-signal obtained through a three-level wavelet packet decomposition.

The energy *e_i_* is calculated as:

(11) $$e_{i}=\sum_{k=1}^{n}{\left|x_{ij}\right|}^{2}$$

where *n* is the number of sampling points of the original signal *S*, and *x_ij_* represents the *j*-th data point of the reconstructed *i*-th sub-band signal.

The total wavelet packet energy *E_wp_* of the time-domain signal is expressed as:

(12) $$E_{wp}=e_{1}+e_{2}+e_{3}+e_{4}+e_{5}+e_{6}+e_{7}+e_{8}$$

## 3. Results and Discussion

### 3.1. Assessment of Sensor Performance

Table 4 summarizes the capacitance variation of six PZT-5A patches under five conditions. The average capacitance progressively decreased from 1.20 nF in Condition 1 (initial state) to 1.06 nF in Condition 2 (epoxy coating), 1.00 nF in Condition 3 (water immersion for 15 min), 0.98 nF in Condition 4 (mortar encapsulation), and reached the minimum value of 0.92 nF in Condition 5 (24 h curing). Overall, the capacitance exhibited a reduction of approximately 22.9% compared with the initial state. The sensors exhibited varying degrees of capacitance reduction (16.77–29.76%), with sensor #4 showing the most pronounced decrease, retaining only approximately 70% of its initial capacitance. This observation indicates that the sensors display individual variability and sensitivity under the influence of external media and encapsulation processes. Such behavior can be attributed to the combined effects of weakened interfacial polarization, differences in the dielectric properties introduced by the encapsulation materials, and polarization effects of water molecules.

**Figure 6:**
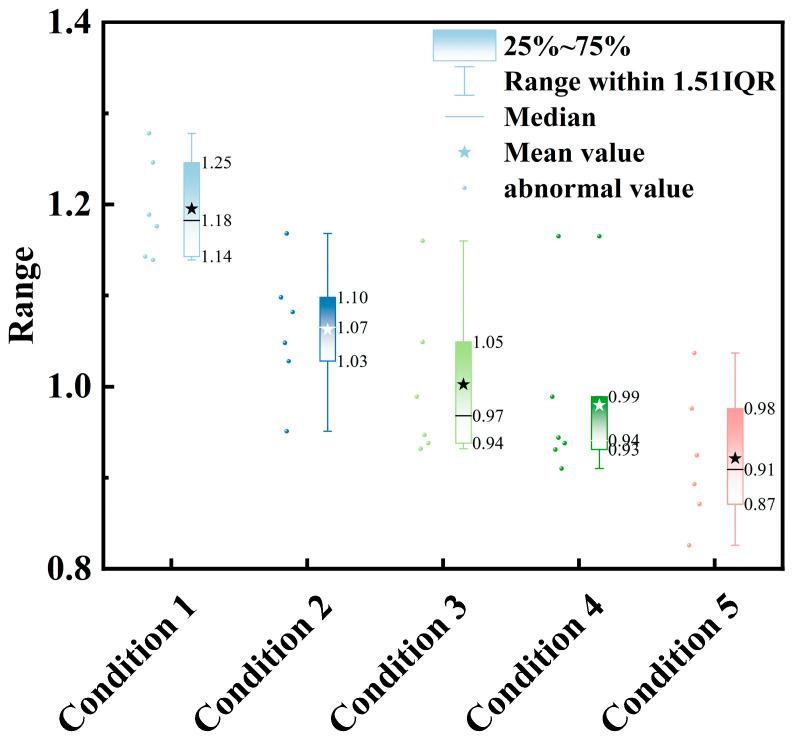
Boxplot of capacitance distribution of PZT-5A under different conditions.

To further illustrate the capacitance distribution, boxplots were used for visualization (Figure 6). The results show that, in Condition 1, the box height was low and the variation among sensors was minimal, indicating good consistency in electrical performance under the initial state. In Conditions 2–3, both the box height and interquartile range increased significantly, reflecting enhanced variability in sensor responses caused by epoxy coating and water immersion. In Condition 4, the overall capacitance decreased and the box slightly narrowed, but the whiskers extended, indicating that most sensors converged in performance while a few remained deviated. Finally, in Condition 5, capacitance further decreased, the box narrowed considerably, and the median was close to the mean, suggesting reduced variability among sensors and stabilized performance after 24 h curing. Overall, the boxplots reveal a progressive decline in capacitance across conditions and illustrate changes in sensor consistency and stability, providing experimental evidence for analyzing the effects of different conditions on the electrical performance of piezoelectric sensors.

**Figure 7:**
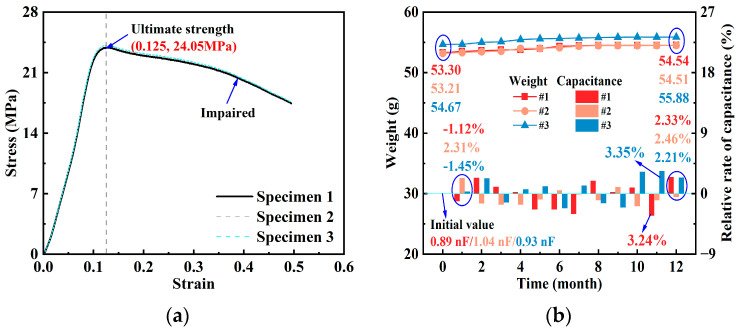
Performance evaluation of the piezoelectric ultrasonic sensor: (**a**) compressive stability; (**b**) waterproof sealing capability.

Figure 7 presents the performance evaluation of the piezoelectric ultrasonic sensors. Figure 7a illustrates the mechanical response of the sensors under uniaxial compression. The stress experienced by the sensors gradually increased with strain, reaching a maximum of 24.05 MPa at a strain of 0.125, at which point the encapsulation provided stable protection for the piezoelectric ceramic. When the strain exceeded 0.125, structural damage occurred, leading to performance degradation. Furthermore, other sensors also exhibited similar mechanical responses under the same testing conditions. These results define the stress–strain limit of the sensors and provide critical experimental guidance for their deployment and encapsulation design in practical engineering applications.

Figure 7b shows the variation in sensor mass and capacitance during long-term water immersion. The sensor mass exhibited a gradual increase over time: sensor # 1 increased from 53.30 g to 54.54 g (2.32% growth), while sensors # 2 and # 3 increased by 1.31 g and 1.21 g, corresponding to growth rates of 2.46% and 2.21%, respectively. This trend is primarily attributed to capillary water absorption of the cement mortar and the slow hydration of residual unhydrated components under immersion conditions.

Despite the slight mass increase during long-term immersion, the capacitance of the sensors remained largely stable after the encapsulation material was fully cured (28 d), showing only minor fluctuations around the initial values. After 1 month of immersion, the capacitances were 0.88, 1.061, and 0.93 nF, with relative changes of −1.12%, 2.31%, and −1.45%; at 12 months, the relative changes were 2.33%, 2.46%, and 2.21%. Overall, the capacitance variations were maintained within ±4%, with a maximum deviation of 3.35% and a minimum of −3.24%. These results indicate that, although long-term water immersion slightly increases the sensor mass, the capacitance performance remains highly stable, demonstrating excellent waterproofing capability of the piezoelectric ultrasonic sensors.

### 3.2. Correlation Analysis Between Piezoelectric Signal Features and Penetration Resistance (0–13 h)

**Figure 8:**
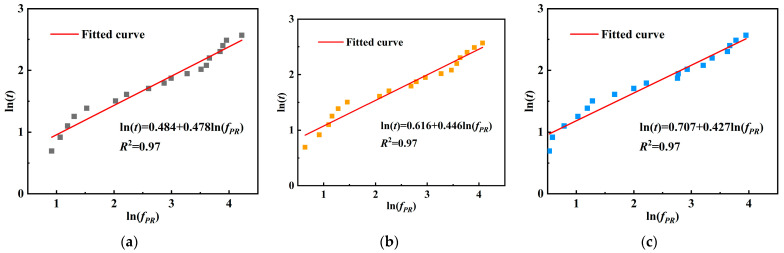
Time–penetration resistance fitting curves: (**a**) *w*/*c* = 0.40; (**b**) *w*/*c* = 0.45; (**c**) *w*/*c* = 0.50.

Table 5 and Figure 8 illustrates the relationship between hydration time and penetration resistance for different *w*/*c* (0.40, 0.45, and 0.50). According to the specification, the times at which the penetration resistance reaches 3.5 MPa and 28 MPa correspond to the initial and final setting times of concrete, marking the transition from a plastic to a solid state. As shown in the fitted curves, the initial setting times increase with higher *w*/*c* ratios, measured as 2.95 h, 3.24 h, and 3.46 h, indicating that lower *w*/*c* ratios accelerate hydration and early structural stiffening. The final setting times also follow this trend, recorded as 7.98 h, 8.19 h, and 8.41 h, suggesting that higher *w*/*c* ratios slow down the hydration process and delay pore structure closure. These results demonstrate that *w*/*c* ratio significantly affects setting behavior, with higher ratios exhibiting delayed structural formation and consequently slower early mechanical development.

**Figure 9:**
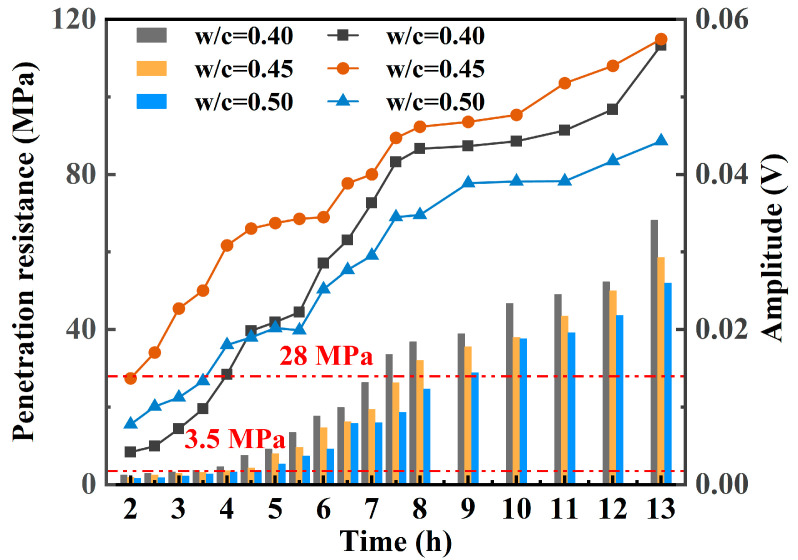
The variation laws of penetration resistance and piezoelectric signal amplitude over time.

Figure 9 illustrates the evolution of piezoelectric signal amplitude and penetration resistance during the 0–13 h hydration period for different water-to-cement ratios. In the initial stage (from casting to initial setting), the concrete remains in a slurry state with numerous interfaces and material heterogeneity, resulting in significant signal attenuation. Consequently, both the piezoelectric signal amplitude and penetration resistance remain low. As the concrete progresses from initial to final setting, hydration accelerates, and hydration products fill the pores and form a continuous skeleton structure. The signal transmission path becomes more defined, leading to a rapid increase in both signal amplitude and penetration resistance, indicating the transition from a plastic to a solid state.

After final setting, the internal structure becomes denser, reducing signal loss and stabilizing the signal amplitude. By 13 h, the piezoelectric signal amplitudes of the *w*/*c* = 0.40, 0.45, and 0.50 specimens reached 76.46%, 80.34%, and 78.54% of their respective final values, with a noticeable decrease in growth rate. At the same time, penetration resistance rose significantly to 68.2 MPa (27.28-fold increase), 58.6 MPa (30.84-fold), and 52.0 MPa (30.59-fold), with corresponding signal amplitudes of 0.0567 V, 0.0575 V, and 0.0443 V, respectively, indicating that early-age strength development was largely completed.

**Figure 10:**
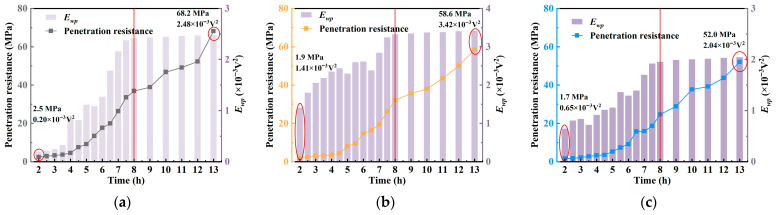
Comparison graph of penetration resistance and WPE curve: (**a**) *w*/*c* = 0.40; (**b**) *w*/*c* = 0.45; (**c**) *w*/*c* = 0.50.

Figure 10 presents the comparative evolution of penetration resistance and WPE over the hydration period under different *w*/*c*. Results indicate that WPE closely follows the early strength development trend of concrete, with both parameters increasing over time. However, unlike the relatively smooth rise in penetration resistance, WPE exhibits more pronounced fluctuations, highlighting its sensitivity to microstructural changes. The variation in penetration resistance primarily reflects the accumulation of the concrete’s macroscopic mechanical strength, whereas WPE captures the signal response characteristics associated with microstructural evolution. Although the temporal trends of the two are somewhat correlated, their changes are not entirely synchronous. This indicates that the initial slope of penetration resistance can serve as a mechanical indicator of early structural evolution, while WPE provides complementary information on microstructural development. Together, they allow for a more comprehensive characterization of the early hydration stages of concrete.

In the early stage (e.g., at 2 h), the concrete remains in a fluid state with a high number of interfaces and significant material heterogeneity. These conditions result in strong ultrasonic signal attenuation and low WPE values, measured as 0.20 × 10^−3^ V^2^, 1.41 × 10^−3^ V^2^, and 0.65 × 10^−3^ V^2^, and 0.65 × 10^−3^ V^2^ for *w*/*c* ratios of 0.40, 0.45, and 0.50, respectively. As hydration progresses, WPE shows a marked increase.

With the transition from a plastic to a solid state, the internal structure becomes denser and more homogeneous, reducing signal attenuation and allowing WPE to increase steadily. By 13 h, the WPE values reach 2.48 × 10^−3^ V^2^, 3.42 × 10^−3^ V^2^, and 2.04 × 10^−3^ V^2^ for the respective *w*/*c* ratios, further confirming its capability to reflect the evolution of hydration and microstructure.

At approximately 8 h, near the final setting time, the WPE values of the three concrete specimens were 2.42 × 10^−3^ V^2^, 3.32 × 10^−3^ V^2^, and 1.95 × 10^−3^ V^2^, respectively. Subsequently, the internal structure of the concrete gradually stabilized, and the energy growth rate noticeably slowed, with WPE approaching saturation. Notably, at this stage, the WPE of all three specimens had exceeded 95% of their respective final saturated values. These results indicate that WPE can serve as an effective indicator for reflecting the evolution of internal concrete structure and strength development, demonstrating strong capability for identifying setting behavior and potential applicability in engineering practice.

### 3.3. Evolutionary Relationship Between Piezoelectric Signal Features and Compressive Strength (1–28 d)

**Figure 11:**
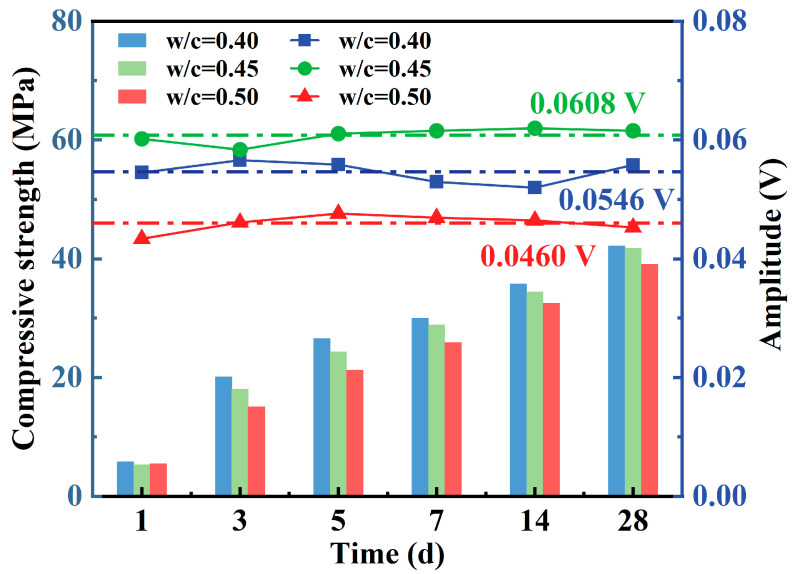
The variation laws of compressive strength and piezoelectric signal amplitude over time.

Table 6 summarizes the compressive strength and piezoelectric signal amplitude of concrete at six typical curing ages: 1, 3, 5, 7, 14, and 28 d. Figure 11 further illustrates their evolution over time. Uniaxial compression tests indicate that compressive strength increases significantly with curing time, exhibiting distinct growth phases. From 1 to 7 d, strength rises rapidly, reaching 48%, 43%, and 39% of the 28-day strength by day 3, and 71%, 69%, and 66% by day 7 for *w*/*c* ratios of 0.40, 0.45, and 0.50, respectively. Between 7 and 14 d, strength continues to increase but at a reduced rate, reaching 85%, 82%, and 83% of the 28-day values. After 14 d, strength growth tends to plateau. Higher *w*/*c* ratios correspond to lower strength at the same age, indicating delayed hydration and reduced early strength development efficiency.

PWPM results show that after 1 day, the increasing material stiffness leads to a gradual rise in signal amplitude, accompanied by some fluctuations. Between 1 and 28 d, the average signal amplitudes are 0.0608 V (*w*/*c* = 0.40), 0.0546 V (*w*/*c* = 0.45), and 0.0460 V (*w*/*c* = 0.50). These differences are attributed to variations in microstructural properties (e.g., porosity and compactness) and the inherent variability in sensor fabrication.

**Figure 12:**
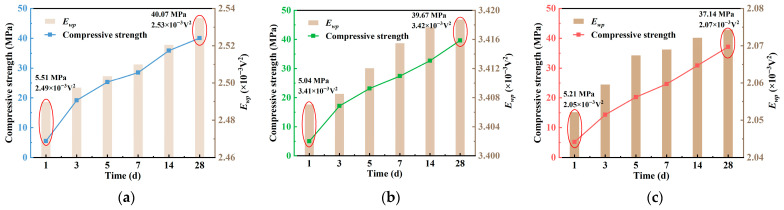
Comparison graph of compressive strength and WPE curve: (**a**) *w*/*c* = 0.40; (**b**) *w*/*c* = 0.45; (**c**) *w*/*c* = 0.50.

To comprehensively characterize the features of piezoelectric signals, a three-level wavelet packet decomposition method was employed to analyze time-domain responses of signals at various curing ages. The reconstructed signals were used to calculate the WPE. Figure 12 illustrates the evolution of WPE for concrete with different water–cement ratios (*w*/*c* = 0.40, 0.45, and 0.50) during the curing period from 1 to 28 d. Results show that WPE increases progressively with curing time and exhibits a strong positive correlation with compressive strength. This trend is attributed to the continuous hydration process, which reduces internal porosity, weakens ultrasonic energy attenuation, and enhances energy received by the sensor. WPE effectively reflects early-stage densification and homogenization of concrete, highlighting its potential for non-destructive evaluation.

Among the three groups, the *w*/*c* = 0.45 group exhibited the highest WPE values across all ages (mean: 3.41 × 10^−3^ V^2^) with the lowest variation (standard deviation: 0.005), suggesting a more uniform and stable internal structure. For the *w*/*c* = 0.40 group, WPE increased from 2.49 × 10^−3^ V^2^ at 1 day to 2.53 × 10^−3^ V^2^ at 28 d, representing a 1.82% increase, indicating continuous improvement in structural compactness. In contrast, the *w*/*c* = 0.50 group showed the lowest average WPE (2.068 × 10^−3^ V^2^) and minimal growth, suggesting that higher porosity resulted in greater energy scattering and attenuation, thereby limiting WPE development.

### 3.4. Identification of Key Hydration Stage Transitions Based on RMSD Analysis

To quantify the stability and variability of piezoelectric signals over different curing ages, the RMSD was introduced. By evaluating the deviation of signal values at each time point from a reference value, RMSD provides insights into the influence of microstructural evolution on piezoelectric response characteristics [29,43].

Considering that piezoelectric signals can reflect material state variations through both time-domain amplitude changes and energy distribution, this study establishes the amplitude-based Root Mean Square Deviation (Amplitude-RMSD) and the WPE-RMSD to quantitatively characterize the evolution of signal amplitude and energy over curing age. The calculation expressions are given as follows:

(13) $$\mathrm{Amplitude-RMSD}\left(t\right)=\sqrt{\frac{1}{N_{t}}\sum_{i=1}^{N_{t}}\frac{{\left(A_{t,i}-A_{0,i}\right)}^{2}}{A_{0,i}^{2}}}$$

(14) $$\mathrm{WPE-RMSD}\left(t\right)=\sqrt{\frac{1}{N_{t}}\sum_{i=1}^{N_{t}}\frac{{\left(E_{t,i}-E_{0,i}\right)}^{2}}{E_{0,i}^{2}}}$$

where *A_t,i_* denotes the signal amplitude of the *i*-th sample at curing age *t*; *A_0,i_* represents the corresponding amplitude at the initial stage; *E_t,i_* and *E_0,i_* are the WPE of the *i*-th sample at curing age *t* and the initial stage, respectively; and *N_t_* is the cumulative number of samples up to curing age *t*.

**Figure 13:**
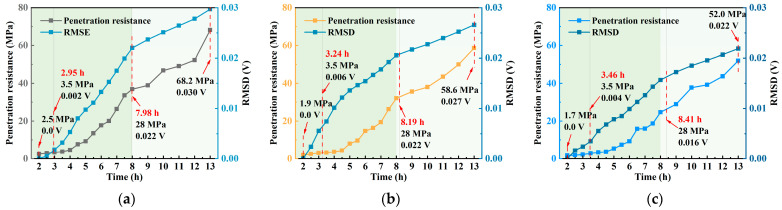
Evolution of Amplitude-RMSD with concrete hydration age from 0 to 13 h: (**a**) *w*/*c* = 0.40; (**b**) *w*/*c* = 0.45; (**c**) *w*/*c* = 0.50.

Figure 13 presents the evolution of Amplitude-RMSD under different *w*/*c* during the early curing period. The overall increasing trend of RMSD with time indicates that as hydration progresses, the internal concrete structure becomes denser, signal fluctuations become more regular, and response stability improves. Specifically, the *w*/*c* = 0.40 group exhibited the fastest RMSD growth, reaching 0.030 V at 13 h, likely due to rapid hydration and early particle interconnection, which induced more frequent signal path perturbations. In contrast, the *w*/*c* = 0.50 group showed the lowest and smoothest RMSD growth, suggesting delayed structural densification. These findings confirm the effectiveness and sensitivity of Amplitude-RMSD in capturing the relationship between signal stability and microstructural evolution.

**Figure 14:**
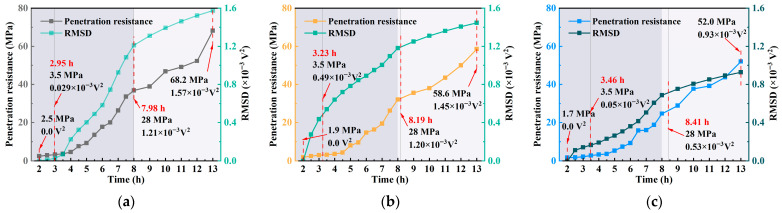
Evolution of WPE-RMSD with concrete hydration age from 0 to 13 h: (**a**) *w*/*c* =0.40; (**b**) *w*/*c* = 0.45; (**c**) *w*/*c* = 0.50.

Figure 14 further illustrates the evolution of the Wavelet Packet Energy-based Root Mean Square Deviation (WPE-RMSD) over the curing age. As the core metric underlying the proposed Energy Deviation Response Index (EDRI), the evolution of WPE-RMSD indicates that this index can sensitively capture the microstructural transitions occurring during the early hydration process of concrete. Using the 2 h energy value as the reference, this metric evaluates the signal energy deviation at different hydration stages. EDRI shows a significant increasing trend, particularly in the early stage (2–5 h), where *w*/*c* = 0.45 exhibits the most pronounced fluctuations. This implies greater flowability and more dynamic changes in internal uniformity, leading to larger signal energy deviations. The *w*/*c* = 0.40 group maintains a relatively high growth rate during the later phase (7–13 h), reflecting sustained densification, whereas the *w*/*c* = 0.50 group shows the slowest increase and a delayed peak, further supporting the notion of slower hydration progression at higher *w*/*c* ratios.

In summary, both Amplitude-RMSD and WPE-RMSD demonstrate strong sensitivity in identifying the final setting stage (around 8 h), indicating their potential for monitoring structural stabilization. However, the recognition of the initial setting stage (2–6 h) is less reliable due to significant signal fluctuations and the absence of distinct turning points-especially in Amplitude-RMSD. This limitation is primarily attributed to the high heterogeneity of the fresh cementitious matrix, where signal propagation is affected by multi-phase interfaces and strong attenuation, and is highly dependent on local microstructure and sensor coupling. As hydration proceeds and a continuous skeleton forms, signal paths stabilize, and piezoelectric responses exhibit clearer evolution patterns.

**Figure 15:**
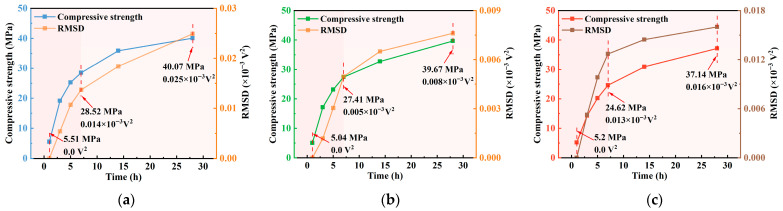
Evolution of WPE-RMSD with concrete hydration age from 0 to 28 d: (**a**) *w*/*c* = 0.40; (**b**) *w*/*c* = 0.45; (**c**) *w*/*c* = 0.50.

Figure 15 illustrates the evolution of EDRI in concrete specimens with different *w*/*c* from 1 to 28 days of curing. It is noteworthy that the amplitude-based metrics used in previous studies exhibited a plateaued trend beyond 1 day, with limited signal fluctuation, thereby making it challenging to capture the effects of mid-to-late-stage structural changes on ultrasonic wave propagation. To enhance sensitivity and discrimination, this study employs the WPE feature as the base variable and calculates its RMSD to construct EDRI, enabling a more accurate capture of the impact of concrete structural evolution on signal response. Using the WPE value at 1 day as a reference, the index reflects deviations over time and can be used to characterize the evolution of signal stability resulting from structural densification during hydration.

During the early curing phase (1–7 days), all three concrete groups displayed a marked increase in EDRI, suggesting rapid structural densification driven by hydration reactions. This densification caused continuous adjustments in ultrasonic propagation paths, leading to greater signal energy variability. Among the groups, the *w*/*c* = 0.40 group exhibited the most pronounced increase, with EDRI reaching 0.014 × 10^−3^ V^2^ at 7 days-higher than the *w*/*c* = 0.45 (0.005 × 10^−3^ V^2^) and *w*/*c* = 0.50 (0.013 × 10^−3^ V^2^) groups. This indicates that the lower *w*/*c* ratio facilitated faster hydration product formation and early development of a solid-phase skeleton, thereby amplifying signal response fluctuations.

In the mid-age phase (7–28 days), the EDRI continued to increase across all groups, albeit at a slower rate. This trend reflects a stabilization of the structural densification process and a gradual convergence of signal fluctuations. The *w*/*c* = 0.40 group reached the highest EDRI value at 28 days (0.025 × 10^−3^ V^2^), indicating the largest overall fluctuation range. In contrast, the *w*/*c* = 0.45 group exhibited the lowest and most stable RMSD value (0.008 × 10^−3^ V^2^), suggesting a more uniform and steady structural development. The *w*/*c* = 0.50 group showed a slower increase during early curing and a slight acceleration in the later stages, with a final EDRI of 0.016 × 10^−3^ V^2^ at 28 days.

In summary, the EDRI metric effectively captures ultrasonic signal variations caused by internal microstructural evolution during both early hydration (0–13 h) and extended curing (1–28 days). Its high sensitivity and differentiation capability across *w*/*c* ratios support its potential as a valuable auxiliary parameter for evaluating concrete setting, structural development, and stability progression.

Based on the above results, the EDRI effectively characterizes the early hydration process of concrete, providing a promising approach for in situ evaluation of structural formation. However, several limitations remain in this study:

(1) The investigation mainly focused on the evolution of PZT signal responses during hydration, with only one specimen tested under each water-to-cement ratio. Although consistent trends were observed, statistical reliability needs further confirmation through additional parallel tests;
(2) Only three typical water-to-cement ratios (0.40, 0.45, 0.50) were considered, without covering a broader range of mix designs or different cementitious systems (e.g., blended cements);
(3) The effects of environmental factors such as temperature, humidity, and structural constraint stresses on the piezoelectric responses were not systematically evaluated;
(4) The analysis was primarily based on piezoelectric signal amplitude and WPE indicators to describe structural evolution, without establishing a quantitative correlation between signal features and macroscopic mechanical properties (e.g., elastic modulus).

Future work will focus on the following aspects:

(i) Conducting multiple parallel tests under identical conditions and expanding the experimental parameter space to systematically examine the influence of material composition on piezoelectric responses and enhance data robustness;
(ii) Investigating the coupled effects of temperature and humidity through controlled laboratory experiments; and
(iii) Employing multi-feature fusion and machine learning approaches to establish predictive relationships between piezoelectric signal characteristics and macroscopic mechanical performance.

## 4. Conclusions

This study employed embedded piezoelectric ultrasonic sensors to conduct in situ monitoring of the concrete hydration process, aiming to identify curing stages based on electromechanical response characteristics. By introducing the Stage-wise Energy Drift Index (SEDI), the limitations of traditional strength-based indicators in early-age monitoring were addressed. The dual functionality of piezoelectric sensors—as both actuators and receivers—was validated, demonstrating excellent response sensitivity and monitoring potential for non-destructive evaluation.

(1) The comprehensive performance evaluation of the piezoelectric ultrasonic sensors indicates that, due to encapsulation processes and interfacial effects, their capacitance values gradually decrease during successive encapsulation steps and eventually stabilize. The mechanical loading tests revealed a maximum load-bearing capacity of 24.05 MPa, providing critical experimental evidence for structural encapsulation design. Long-term water immersion tests showed that although the sensor mass increased slightly over time, the capacitance variation consistently remained within ±4%, demonstrating excellent electrical stability and waterproof performance. Overall, the sensors exhibited robust stability and reliability under diverse environmental conditions, providing strong experimental support for their potential use in future studies of structural monitoring.
(2) During the early hydration stage (0–13 h), both the piezoelectric signal amplitude and WPE exhibited strong responsiveness to the microstructural evolution of concrete. A lower water-to-cement ratio (*w*/*c* = 0.40) significantly accelerated the hydration process, with initial and final setting times (corresponding to 3.5 MPa and 28 MPa) recorded at 2.95 h and 7.98 h, respectively—noticeably earlier than those in the higher *w*/*c* group. Both amplitude and WPE effectively captured the critical transition from slurry to solid state. Compared with the relatively stable penetration resistance, WPE showed greater sensitivity to microstructural changes such as porosity and interfacial density, exhibiting notable fluctuations. After final setting (~8 h), WPE approached saturation (reaching over 95% of its final value), indicating that WPE can reliably reflect the process of concrete setting and the formation of early strength, and providing experimental support for its potential use in related monitoring studies.
(3) From 1 to 28 d, the signal amplitude exhibited a gradual increase with slight fluctuations, ranging from 0.0460 to 0.0608 V. However, WPE was more sensitive to the densification process, effectively reflecting the internal microstructural reconstruction driven by hydration. Based on the measured WPE values, the *w*/*c* = 0.45 group consistently exhibited the highest and most stable energy values across all curing ages, suggesting relatively better structural uniformity. In contrast, specimens with higher *w*/*c* ratios showed lower energy responses, which can be attributed to higher porosity and increased signal attenuation.
(4) The EDRI derived from piezoelectric signal responses demonstrated reliable stage recognition capabilities in monitoring the structural evolution of concrete. It effectively characterized structural disturbances induced by early hydration (0–13 h) and tracked the densification process during the 1–28 d period, offering good temporal continuity. Although EDRI performed well in identifying the final setting and later stages, its accuracy was limited during the initial setting phase (approximately 2–6 h), due to unstable signal fluctuations caused by paste fluidity and interfacial heterogeneity. Overall, as a statistical indicator of signal deviation derived from experimental observations, EDRI provides quantitative insight into key transitional phases in the transformation of concrete from rheological to solid states.

## Figures and Tables

**Table 1:** Mix Proportion Design.

Number	Cement (kg/m^3^)	Water (kg/m^3^)	*w*/*c*	Sand (kg/m^3^)	Gravel (kg/m^3^)	Water Reducing Agent (kg/m^3^)
1	361	170	0.40	763	1053	3.78
2	321	170	0.45	799	1059	3.75
3	289	170	0.50	832	1059	3.75

**Table 2:** Material properties of PZT-5A.

Material Property		Value	Material Property		Value
Bulk density (g/cm^3^)	*ρ*	7.6	Young’s modulus (10^9^ N/m^2^)	${Y^{E}}_{11}$	56
Poisson’s ratio	*δ*	0.36	Acoustic impedance (MRayl)	*Z*	22.42
Dielectric constant	$\varepsilon_{r1}^{T}$	3800	Curie temperature (°C)	T_c_	220
	$\varepsilon_{r3}^{T}$	3400	Frequency constant (Hz·m)	*N*_1_	1400
Electromechanical coupling coefficient	*k*_31_	0.36		*N*_3_	1850
	*k*_33_	0.8		*N*_5_	1200
	*k*_15_	0.68	Ultrasonic velocity (m/s)	$V_{dn}^{E}$	2950
Piezoelectric strain constant (pC/N)	*d*_31_	−280		$V_{1n}^{E}$	2900
	*d*_33_	650		$V_{3n}^{E}$	3600
	*d*_15_	860		$V_{tn}^{E}$	3930

**Table 3:** Reference for probe size selection.

*f_PR_* (MPa)	0.2~3.5	3.5~20.0	20.0~28.0
*A* (mm^2^)	100	50	20

**Table 4:** Capacitance measurements of PZT-5A under different conditions.

	Condition	Capacitance (nF)					Relative Error (%)
Number		1	2	3	4	5	
#1		1.14	0.95	0.95	0.94	0.89	21.60
#2		1.25	1.17	1.16	1.17	1.04	16.77
#3		1.19	1.03	0.93	0.93	0.93	22.20
#4		1.18	1.05	0.94	0.91	0.83	29.76
#5		1.28	1.08	1.05	0.99	0.98	23.63
#6		1.14	1.10	0.99	0.94	0.87	23.80
Mean value		1.20	1.06	1.00	0.98	0.92	22.91

**Table 5:** The variation laws of penetration resistance and piezoelectric signal amplitude over time.

Time (h)	Penetration Resistance (MPa)			Amplitude (V)		
	*w*/*c* = 0.4	*w*/*c* = 0.45	*w*/*c* = 0.5	*w*/*c* = 0.4	*w*/*c* = 0.45	*w*/*c* = 0.5
2	2.5	1.9	1.7	0.0042	0.0137	0.0078
2.5	2.9	2.5	1.8	0.0049	0.0170	0.0101
3	3.3	3	2.2	0.0072	0.0227	0.0113
3.5	3.7	3.2	2.8	0.0098	0.0250	0.0134
4	4.6	3.6	3.3	0.0142	0.0309	0.0180
4.5	7.6	4.3	3.6	0.0198	0.0330	0.0190
5	9.2	8	5.3	0.0210	0.0337	0.0202
5.5	13.5	9.6	7.4	0.0222	0.0343	0.0199
6	17.7	14.7	9.2	0.0286	0.0345	0.0252
6.5	20	16.3	15.8	0.0315	0.0389	0.0277
7	26.4	19.4	16	0.0363	0.0400	0.0296
7.5	33.6	26.3	18.7	0.0416	0.0447	0.0345
8	36.9	32.1	24.7	0.0433	0.0462	0.0348
9	38.9	35.6	28.9	0.0437	0.0468	0.0389
10	46.8	38	37.7	0.0443	0.0477	0.0391
11	49.1	43.5	39.2	0.0457	0.0518	0.0391
12	52.3	50	43.7	0.0484	0.0540	0.0417
13	68.2	58.6	52	0.0567	0.0575	0.0443

**Table 6:** The variation laws of compressive strength and piezoelectric signal amplitude over time.

Time (h)	Compressive Strength (MPa)			Amplitude (V)		
	*w*/*c* = 0.40	*w*/*c* = 0.45	*w*/*c* = 0.50	*w*/*c* = 0.40	*w*/*c* = 0.45	*w*/*c* = 0.50
1	5.8	5.31	5.48	0.0545	0.0602	0.0434
3	20.16	18.05	15.08	0.0566	0.0584	0.0461
5	26.62	24.37	21.29	0.0559	0.0611	0.0476
7	30.02	28.85	25.92	0.0529	0.0616	0.0469
14	35.76	34.42	32.51	0.0520	0.0620	0.0465
28	42.18	41.76	39.09	0.0558	0.0616	0.0453

## Data Availability

The original contributions presented in this study are included in the article. Further inquiries can be directed to the corresponding authors.
